# Effect of entrepreneurial education and creativity on entrepreneurial intention in college students: mediating entrepreneurial inspiration, mindset, and self-efficiency

**DOI:** 10.3389/fpsyg.2023.1240910

**Published:** 2023-09-15

**Authors:** Yue Li, Keyan Cao, Hashem Salarzadeh Jenatabadi

**Affiliations:** ^1^Department of Environmental Design, Faculty of Art and Design, Shandong Women’s University, Jinan, Shandong, China; ^2^Institute of Economic and Social Development, Shenzhen Polytechnic University, Shenzhen, Guangdong, China; ^3^Department of Science and Technology Studies, Faculty of Science, Universiti Malaya, Kuala Lumpur, Malaysia

**Keywords:** entrepreneurial intention, entrepreneurial inspiration, entrepreneurial creativity, structural equation modeling, entrepreneurial mindset, entrepreneurial self-efficiency

## Abstract

Entrepreneurship in higher education is increasingly valuing entrepreneurial creativity as a significant driver for improving university students’ innovative abilities. The purpose of this study was to examine the direct influence of entrepreneurial education and creativity on entrepreneurial intention, as well as the indirect role of entrepreneurial inspiration, mindset, and self-efficiency. This study gathered survey responses from 448 university business students from three Chinese provinces of Shandong, Jiangsu and Zhejiang. The results indicated that entrepreneurial education and creativity have a positive and significant effect on entrepreneurial intent. In addition, the results demonstrated that the combination of entrepreneurial mindset, inspiration, and self-efficacy partially mediates the relationship between entrepreneurial education and entrepreneurial creativity. In addition, additional implications and restrictions are discussed in this article.

## 1. Introduction

Entrepreneurship plays a significant role in driving economic growth and creating a positive impact on the economy (Värlander et al., 2020). Entrepreneurs are known for their ability to identify and exploit market gaps (Yi and Journal, 2021). They develop innovative products, services, and processes, which lead to technological advancements. This, in turn, improves productivity, efficiency, and competitiveness within industries, driving economic growth (Neneh, 2022). According to the social cognitive theory developed by Bandura (1992), an individual’s level of self-efficacy can be increased through the pursuit of entrepreneurial education. Individuals are given the opportunity to engage in entrepreneurial activities such as spotting opportunities, doing company feasibility analyses, and putting together actionable business plans as a result of this. Previous research has noted that entrepreneurship education, an entrepreneurial mindset, and creativity cultivate young talents and develop entrepreneurial intention among individuals to become entrepreneurs (Cui and Bell, 2022; Sun et al., 2023; Yan et al., 2023). According to these studies, with a rising number of university graduates, effective job hunting has become a critical challenge in China’s higher education system. At both the individual and the societal level, it has been argued that there are benefits connected with entrepreneurship, such as the creation of self-employment opportunities, improvements in living standards, and reductions in poverty, along with other forms of social and economic growth. According to Israr and Saleem (2018), students nowadays are showing a preference for self-employment, which has contributed to the rise in popularity of entrepreneurship as a vocation among students all over the world in recent years. The entrepreneurial mindset refers to a specific set of attitudes, behavior, and ways of thinking that are commonly associated with successful entrepreneurs (Bosman and Fernhaber, 2021). Developing an entrepreneurial mindset is not limited to those who are starting their own businesses. It can also be valuable in corporate settings, where individuals can apply entrepreneurial thinking to drive innovation and problem-solving. Rodriguez and Lieber (2020) believe that, an entrepreneurial mindset is characterized by the capacity to recognize and capitalize on opportunities in the entrepreneur’s sector. According to some theories (Mukhtar et al., 2021; Saadat et al., 2022), the environment can help people develop their mindsets through instruction or experience, which supports the importance of entrepreneurship education.

Entrepreneurial inspiration is a powerful force that motivates individuals to pursue entrepreneurial intention. It acts as a starting point for entrepreneurial journeys, guiding individuals towards identifying opportunities, developing ideas, and ultimately taking action to start their own ventures (Van Ewijk et al., 2021). A positive association has been shown between entrepreneurial intention and the development of entrepreneurial inspiration for the establishment of novel business enterprises (Yu and Lu, 2023). Entrepreneurial inspiration serves as a source of motivation and aspiration for individuals. It sparks a desire within them to pursue their own entrepreneurial ventures and make a positive impact in the business world. This motivation translates into a higher intention to engage in entrepreneurial activities. Entrepreneurial inspiration exposes individuals to new ideas, business models, and market opportunities (Kirzner, 2019). It expands their perception of potential entrepreneurial opportunities and widens their horizons. This increased awareness of possibilities encourages individuals to consider entrepreneurship as a viable career option and increases their intention to seize those opportunities (Yu and Lu, 2023). The relationship entrepreneurial inspiration, education, and intention were considered previous studies (Cui et al., 2021; Dana et al., 2021; Wang et al., 2022). Less is known about the direct and indirect relationships between entrepreneurial education, entrepreneurial inspiration, and entrepreneurial mindset in the context of Chinese student entrepreneurial intention.

This research makes a contribution to the social cognitive theory developed by Bandura (1985), which helps explain individual self-efficacy and how it contributes to the growth of entrepreneurs. Entrepreneurial self-efficacy is an important psychological factor that shapes an individual’s entrepreneurial intentions, actions, and outcomes (Setiawan, 2014). It influences their willingness to pursue entrepreneurial opportunities, persist in the face of challenges, and perform at their best. Furthermore, experts argued that knowing entrepreneurial self-efficacy is critical, particularly when it comes to starting, managing, and developing a new business (Yang et al., 2023). Individuals who felt a high level of self-efficacy will hence have more cognitive minds. Jiatong et al. (2021a) pointed out that self-efficacy is a social-cognitive process that develops individuals’ cognitive mindsets in the form of entrepreneurial intention and entrepreneurial behavior.

We contribute to the emerging body of knowledge on this essential topic in two ways with this article. First, the research presents empirical evidence on the influence of entrepreneurial inspiration, self-efficiency, creativity, and mindset on the intention to become an entrepreneur in China, where entrepreneurship education is in its early stages. The second addition of this study is our emphasis on three mediators (entrepreneurial inspiration, entrepreneurial self-efficacy, and entrepreneurial mindset) in the link between entrepreneurial education and entrepreneurial intention.

The article is divided into four sections. The first section provides an introduction, which is a statement of the problem and objectives of the study. The second section is about the review of existing literature and the development of hypotheses. The third section describes the research technique. The final section delves into the empirical data and discusses the findings. Finally, we make several suggestions to promote university students’ entrepreneurial intentions.

## 2. Literature review and hypotheses development

According to Beauchamp et al. (2019) research, the term “social cognitive theory” refers to a learning theory that places an emphasis on self-efficacy, modeling, and observational learning of the individual. According to this hypothesis, individuals are more likely to follow their goals if they believe that their talents and abilities are capable of achieving the intended outcomes (Mensah et al., 2023). This was seen by the researchers that developed this theory. Individuals are able to improve their social cognition, consistently govern their ideas, and make their entrepreneurial acts more directional, logical, and substantial with the assistance of educational programmes that focus on entrepreneurship. This research makes use of the social cognitive theory to investigate how students who have a high degree of entrepreneurial education, an entrepreneurial attitude, and creativity increase their capability to create entrepreneurial self-efficacy, which in turn influences entrepreneurial intention (Yu and Lu, 2023).

### 2.1. Impacts of entrepreneurial education on entrepreneurial intention

Entrepreneurial intention has been highlighted in examinations of its association with entrepreneurship education (Díaz-García and Jiménez-Moreno, 2010; Ramadani et al., 2022). According to Alshebami and Al Marri (2022), this is because entrepreneurial intention is the best predictor of entrepreneurial behavior. According to Pan et al. (2022), entrepreneurial education plays a critical part in determining both attitudes toward and intentions regarding starting a business. Fu et al. (2022) found that previous studies felt that entrepreneurial education has an essential role in increasing the talents of the Chinese students, which in turn drives business activity. In addition, empirical research has established the processes by which entrepreneurship education improves entrepreneurial intentions. These mechanisms can be found in educational programs that focus on entrepreneurship. Education in entrepreneurship enables individuals to make do with fewer resources by facilitating the sharing of relevant information and knowledge in an organized fashion. According to Wardana et al. (2020) research, persons who have an interest in learning about entrepreneurship are more likely to engage with their contemporaries and fellows, as well as create an entrepreneurial image. These types of educational models not only stimulated the students’ intention to study entrepreneurship but also prompted the students to act on their intention to become entrepreneurs (Salamzadeh A et al., 2022). The fundamental function of entrepreneurial education is the enhancement of entrepreneurial knowledge, skill, and attitude. On the basis of these studies, we concluded that individuals who perceive a high level of entrepreneurial education are more likely to pursue an entrepreneurial career. Accordingly, our first hypothesis is based on findings demonstrating the correlation between entrepreneurship education and entrepreneurial behavior (Sun et al., 2023):

*H1:* Entrepreneurship education has a significant positive impact on entrepreneurial intention.

### 2.2. Impacts of entrepreneurial education on entrepreneurial inspiration

Entrepreneurship education helps individuals explore and identify their areas of interest and passion. By exposing students to a wide range of business concepts, industries, and entrepreneurial opportunities, education can help individuals discover what resonates with them personally. This self-discovery process can provide direction and inspiration for aspiring entrepreneurs to pursue their entrepreneurial ideas (Bouncken, 2018; Salamzadeh Y et al., 2022). Entrepreneurship education equips individuals with knowledge, skills, and practical tools required for entrepreneurship. The acquisition of this knowledge can boost the confidence of aspiring entrepreneurs, making them feel more prepared and capable of starting their own ventures. Increased confidence can fuel inspiration and motivation, as individuals believe in their abilities and are more willing to take calculated risks. Entrepreneurship education provides the knowledge, skills, and practical experiences necessary for entrepreneurship, while entrepreneurial inspiration provides the motivation, passion, and drive to embark on the entrepreneurial journey. The combination of education and inspiration can empower individuals to pursue their entrepreneurial aspirations and increase their chances of entrepreneurial success. Therefore, we considered the following hypothesis:

*H2:* Entrepreneurship education has a significant positive impact on entrepreneurial inspiration.

### 2.3. Impacts of entrepreneurial education on entrepreneurial mindset

The entrepreneurial mindset promotes a commitment to lifelong learning and continuous personal and professional growth. Individuals with an entrepreneurial mindset understand that entrepreneurship is an evolving journey that requires constant adaptation and learning. They actively seek out new knowledge, stay updated on industry trends, and remain open to new ideas and perspectives. This mindset aligns with the ongoing nature of entrepreneurial education, encouraging individuals to continue their learning journey beyond formal educational experiences. As overall, entrepreneurial education plays a vital role in shaping and nurturing the entrepreneurial mindset. It provides individuals with the necessary knowledge, skills, experiences, and support systems to develop the attitudes, behaviors, and thinking patterns that are essential for successful entrepreneurship. Based on scientific evidence, an entrepreneurial mindset strongly correlates with an individual’s entrepreneurial behavior, directing behavioral patterns toward entrepreneurial activities and outcomes (Ratten and Jones, 2021). Education therefore has the power to influence attitude, which in turn forecasts entrepreneurial intention. The following hypotheses were defined based on the aforementioned statement:

*H3:* Entrepreneurship education has a significant positive impact on entrepreneurial mindset.

### 2.4. Impacts of entrepreneurial mindset on entrepreneurial intention

According to Kuratko et al. (2021), an entrepreneurial mindset is an individual’s dedication to engaging in entrepreneurial activity. According to Fernando and Nishantha (2019), an entrepreneurial mentality is an individual’s propensity to take risks, have a need for achievement, and be passionate about starting a new firm, as well as the ability to design, plan, and manage projects to attain entrepreneurial goals. The term “entrepreneurial intention” refers to the conscious decision to pursue a new line of work. In addition, research has linked the desire to be an entrepreneur to the desire to seek out, evaluate, and capitalize on possibilities with the aid of strategy, organization, procedure, and resources. An entrepreneurial mindset is directly linked to an individual’s entrepreneurial behavior, according to research evidence (Sun et al., 2023). This mindset orients behavioral patterns toward activities and outcomes relevant to entrepreneurship. Therefore, education has the capacity to meld one’s attitude, which may subsequently be used to forecast an intention to engage in entrepreneurial activity. Several studies have investigated the relationship between entrepreneurial mindset and entrepreneurial intention (Ashourizadeh et al., 2014; Chang et al., 2022; Cui and Bell, 2022). Abdelwahed and Alshaikhmubarak (2023) conducted research on the entrepreneurial mindsets of vocational students in Iran and discovered that entrepreneurial mindset had a positive and significant impact on entrepreneurial intention. Using a sample of 494 university students from China, Yan et al. (2023) conducted a study on the entrepreneurial mindset and entrepreneurial intention. They discovered a positive correlation between the entrepreneurial mindset and the intention to pursue an entrepreneurial career. In the meantime, Jung and Lee (2020) conducted a study on the entrepreneurial mindsets of college students in South Korea in order to predict the students’ intentions to start their own businesses. The study’s findings indicate that entrepreneurial traits such as innovativeness, autonomy, and pro-activeness positively developed the entrepreneurial mindset of students who went on to start their own businesses. These studies have consistently found that individuals with a stronger entrepreneurial mindset are more likely to have higher entrepreneurial intentions. A key aspect of the entrepreneurial mindset is the ability to recognize and exploit opportunities. Individuals with an entrepreneurial mindset are more likely to identify potential business opportunities and envision themselves as entrepreneurs, leading to stronger entrepreneurial intentions. These earlier investigations have led us to the conclusion that people with entrepreneurial mindsets engage in entrepreneurial activities more actively than other individuals. As an outcome, we hypothesised that:

*H4:* Entrepreneurial mindset has a significant positive impact on entrepreneurial intention.

### 2.5. Impacts of entrepreneurial creativity on entrepreneurial intention

Entrepreneurial creativity refers to the ability of entrepreneurs to generate novel and innovative ideas, solutions, and approaches in the context of starting and managing their ventures. Entrepreneurs with strong creative abilities excel in generating new and original ideas. They can identify opportunities that others may overlook and come up with innovative solutions to address market needs or problems. This creativity in idea generation is a fundamental aspect of entrepreneurship. Entrepreneurial creativity is a valuable asset for entrepreneurs as it enables them to differentiate themselves in the marketplace, identify unique business opportunities, and develop innovative solutions (Diawati et al., 2023). It has been suggested by previous studies, such as Shi et al. (2020) and Kumar and Shukla (2022), that creativity is particularly important for entrepreneurial efforts, and that entrepreneurial endeavours themselves are creative endeavours. Entrepreneurial creativity is often driven by passion and intrinsic motivation. Individuals with creative inclinations have a strong desire to explore, innovate, and bring their unique ideas to life. This motivation and passion contribute to the development and maintenance of entrepreneurial intention as they are inspired to pursue their creative ideas through entrepreneurship (Hu et al., 2018). Shi et al. (2020) used a survey of 523 university students in China to study the relationship between creativity and the theory of planned behavior on entrepreneurial intention. They found that people with a high level of creativity are more likely to have a positive attitude and high self-belief in entrepreneurial activities. Therefore, creativity serves as a catalyst for entrepreneurial intention and contributes to the development of unique and innovative ventures. Compared to the abovementioned set of research, the vast majority of earlier investigations discovered a favourable association between creative intention and entrepreneurial intention. Therefore, we hypothesised that creative thinking would favourably contribute to an intention to engage in entrepreneurial activity.

*H5:* Entrepreneurial creativity has a significant positive impact on entrepreneurial intention.

### 2.6. Impacts of entrepreneurial inspiration on entrepreneurial intention

Entrepreneurial inspiration can boost an individual’s confidence and belief in their ability to succeed as an entrepreneur. Being inspired by role models or success stories, or having a strong passion for a specific area, instils a belief that they can make a positive impact and overcome challenges. This self-confidence and belief enhance their entrepreneurial intention by strengthening their conviction in their ability to pursue and succeed in entrepreneurship. According to Souitaris et al. (2007), the emotional support that comes in the form of “triggers” that originates from either verbal persuasion or positive encouragement on an entrepreneurial profession has a beneficial effect on an individual’s perspective of an entrepreneurial career. The shift in one’s heart and/or mind can be very powerful, which might lead to a change in one’s intentions regarding entrepreneurship. Entrepreneur ship inspiration events or inputs can be considered as contextual supports having a direct effect on the professional decision-making process (Wang et al., 2022). According to Souitaris et al. (2007) empirical study of engineering students, entrepreneurship inspiration has a stronger positive relationship with entrepreneurial intention than resources, knowledge transfer, or other educational advantages. This understanding comes from the social-cognitive career theory perspective, which is applied to the context of entrepreneurship.

*H6:* Entrepreneurial inspiration has a significant positive impact on entrepreneurial intention.

### 2.7. The mediating roles of entrepreneurial mindset, inspiration, and self-efficiency

The entrepreneurial mindset can indeed serve as a mediator between entrepreneurial education and entrepreneurial intention.

Several early studies (Mukhtar et al., 2021; Saadat et al., 2022) have shown that an entrepreneurial mindset is a way of thinking that makes people act in ways that are linked to entrepreneurship and its culture and output. These researchers say that having an entrepreneurial attitude is related to how people think. Yan et al. (2023) agree with this point of view and confirm that the entrepreneurial mindset gives possible insights into some results that are needed for entrepreneurial studies. An entrepreneurial mindset enables individuals to connect with and internalize the knowledge and skills acquired through entrepreneurial education. It helps individuals understand the practical implications of the education, apply it in real-world scenarios, and recognize opportunities for entrepreneurial action. Developing an entrepreneurial mindset helps individuals overcome the barriers and challenges associated with entrepreneurship. It strengthens their belief in their own abilities, increases their confidence, and reduces the fear of failure. As a result, individuals with an entrepreneurial mindset are more likely to translate their entrepreneurial education into entrepreneurial intentions. Thus, this research intends to have the following hypothesis:

*H7:* Entrepreneurial mindset is a mediator in the relationship between entrepreneurial education and entrepreneurial intention.

Entrepreneurial inspiration, which grows and develops as a result of learning about beliefs and entrepreneurship, has a big impact on the decision to start a business. Cui et al. (2021) say that inspiration is an emotional factor that gets people excited and moving towards their goals. Entrepreneurial inspiration is a change of heart and mind that is caused by a set of events or educational programmes that are designed to help people become entrepreneurs (Wang et al., 2022). Wibowo et al. (2022) say that entrepreneurship schooling gives people ideas. Entrepreneurial education often provides individuals with theoretical knowledge and practical skills related to entrepreneurship. However, the presence of inspiring entrepreneurial stories, case studies, or interactions with successful entrepreneurs can make the education more engaging, relevant, and relatable. Entrepreneurial inspiration bridges the gap between educational content and real-life examples, thereby increasing the perceived value and interest in entrepreneurial education. Entrepreneurial inspiration enhances the likelihood that individuals who receive entrepreneurial education will develop a strong intention to become entrepreneurs.

*H8:* Entrepreneurial inspiration is a mediator in the relationship between entrepreneurial education and entrepreneurial intention.

**Figure 1:**
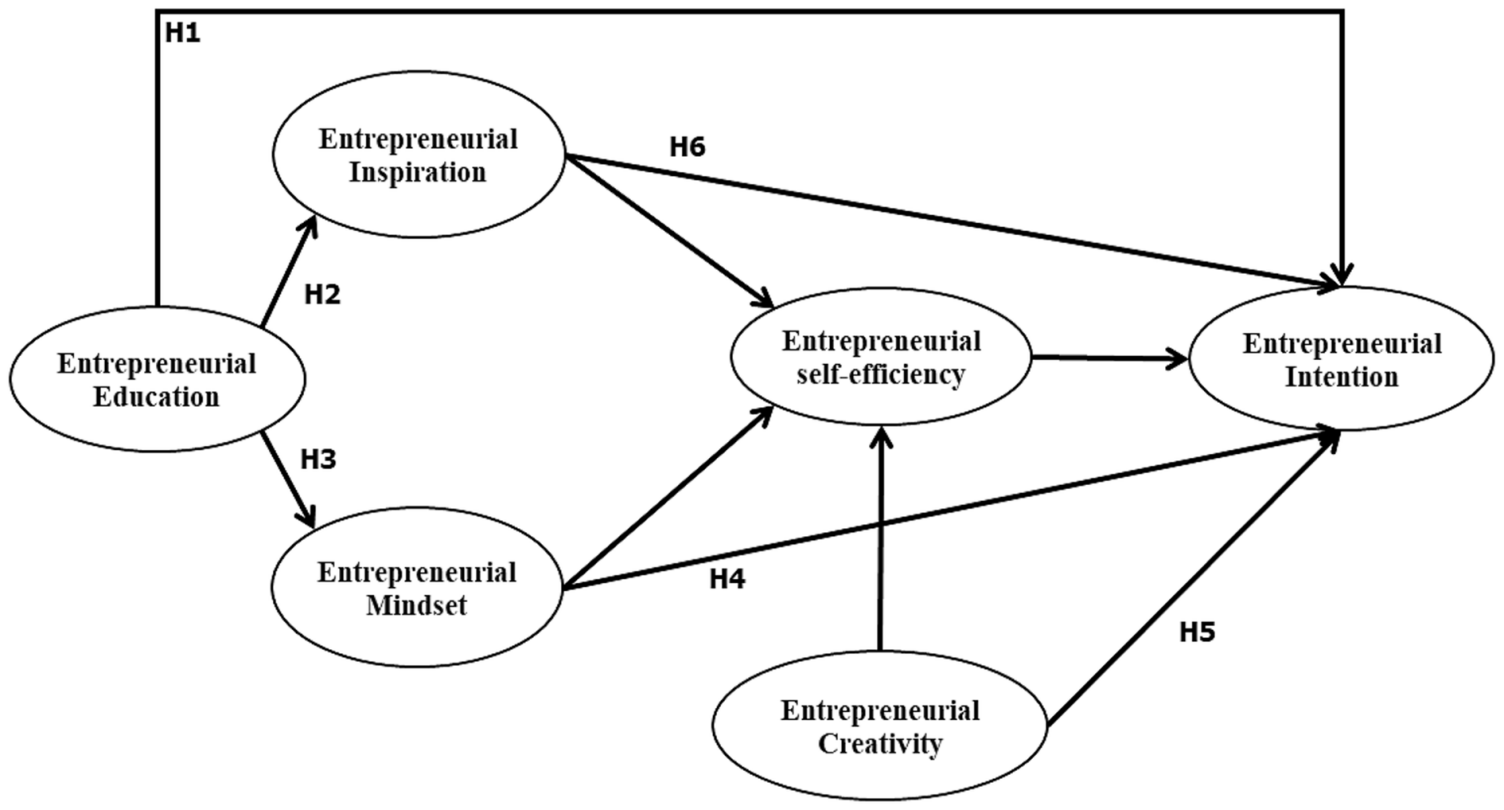
Research framework.

According to Barbaranelli et al. (2019), self-efficacy is the belief that one has in oneself to complete actions that are goal-oriented. Self-efficacy is also linked to people’s propensity to pursue their own objectives. According to a previous study (Wray et al., 2022), self-efficacy has a significant role in determining an individual’s entrepreneurial intention and behavior. Entrepreneurial self-efficacy refers to an individual’s belief in their own capabilities to successfully perform entrepreneurial tasks and overcome challenges (Gielnik et al., 2020). Additionally, a growing number of studies in the fields of entrepreneurship and social psychology have discovered that self-efficacy plays a substantial mediating role as a direct and indirect variable. Self-efficacy, according Li et al. (2020), is the key element that influences a person’s behavior through their cognitive processes, proactive personality, and result expectations. In addition, academics believe that entrepreneurial self-efficacy significantly influences an individual’s decision to pursue entrepreneurial opportunities. Those who have higher levels of self-efficacy are more likely to believe that they can overcome challenges and succeed in their ventures (Al-Qadasi et al., 2023). Individuals with higher levels of entrepreneurial self-efficacy are more likely to believe in their ability to start and manage their own venture successfully. This confidence boosts their entrepreneurial intention by making them more inclined to pursue entrepreneurial opportunities (Ferreira-Neto et al., 2023). The entrepreneurial mindset fosters a sense of self-confidence and self-belief, which contributes to higher levels of entrepreneurial self-efficacy (Aima et al., 2020). When individuals have higher levels of entrepreneurial self-efficacy, they perceive themselves as competent and capable of successfully starting and managing a venture. This belief in their abilities boosts their entrepreneurial intention by increasing their motivation and commitment to pursue entrepreneurial endeavours. Entrepreneurial self-efficacy serves as a mediator in this relationship by explaining how the entrepreneurial mindset influences entrepreneurial intention. The impact of an entrepreneurial mindset on entrepreneurial intention is partially explained by the level of entrepreneurial self-efficacy an individual possesses. Individuals with higher levels of entrepreneurial self-efficacy are thus more likely to perceive higher levels of entrepreneurial education, entrepreneurial inspiration, and entrepreneurial mindset. As a result, the following hypotheses were proposed (see Figure 1):

*H9:* Entrepreneurial self-efficiency is a mediator in the relationship between entrepreneurial education and entrepreneurial intention.

*H10:* Entrepreneurial self-efficiency is a mediator in the relationship between entrepreneurial inspiration and entrepreneurial intention.

*H11:* Entrepreneurial self-efficiency is a mediator in the relationship between entrepreneurial mindset and entrepreneurial intention.

## 3. Materials and methods

### 3.1. Measure

This investigation utilized measurement scales that had been tested and validated by earlier researchers. Students’ responses were evaluated using a 5-point Likert scale ranging from 1 (strongly disagree) to 5 (strongly concur). Table 1 and Appendix A1 present an overview of the theoretical literature and research questions pertaining to latent variables.

**Table 1:** Theoretical literature for measuring latent variables and number of questions.

Latent variable	Number of questions	Theoretical resource
Entrepreneurial education	6 questions	Wardana et al. (2020)
Entrepreneurial mindset	6 questions	Wardana et al. (2020)
Entrepreneurial inspiration	5 questions	Souitaris et al. (2007)
Entrepreneurial self-efficacy	7 questions	Lee and Bobko (1994)
Entrepreneurial creativity	6 questions	Biraglia and Kadile (2017)
Entrepreneurial intention	6 questions	Liñán and Fayolle (2015)

### 3.2. Pilot survey and sampling technique

A pilot study was conducted by administering 100 surveys to business majors at institutions in Shandong, Jiangsu and Zhejiang, China. There were 86 valid responses for 86% response rate.

The primary objective of this study was to examine the entrepreneurial intention of business students, as there has been a noticeable increase in the engagement of university students in the establishment of new businesses within the field of entrepreneurship (Agu, 2021). The pilot survey results indicated that the measuring constructs had satisfactory reliability and validity. A power analysis using the G*Power software found that the study requires a minimum sample size of 421 individuals if the effect size is expected to be 0.15, the alpha value is assumed to be 0.05, and the power is assumed to be 0.80. Convenience sampling was used in this study, and the survey was fielded between March 3 and June 30, 2022. The questionnaires were originally written in English, and the translation was double-checked by two native speakers of Chinese and English utilizing a translation and back-translation method. We also sent out 500 paper-and-pencil surveys to respondents, of which 463 were returned for a response rate of 92.6%. We eliminated 15 questions due to missing information, bringing the total number of replies to 448.

Of the replies received, 58.2% were identified as male and 41.8% were identified as female. The distribution of students in the study sample was as follows: 41.36% were undergraduate students, 32.33% were master’s students, 22.5% were diploma and other students, and 3.81% were PhD students. A majority of the participants (71%) reported having a familial history of entrepreneurship, whereas a minority (29%) did not have such a background. The age categories observed in the study were categorised as follows: 18–25 years old, accounting for 36.4% of the participants; 26–35 years old, representing 41.09% of the sample; 36–45 years old, comprising 16.36% of the respondents; and individuals aged 45 and above, making up 6.21% of the population. In relation to the distribution of majors, the School of Public Administration accounted for 16.5%, the School of Management accounted for 39.45%, the School of Economics accounted for 12.81%, and the School of Finance accounted for 31.24%.

## 4. Results

The primary statistical methods employed in this study were Structural Equation Modeling (SEM) and mediation analysis. SEM is a statistical technique used to analyze complex relationships between observed and latent (unobservable) variables. It’s commonly employed in social sciences, psychology, economics, and other fields to examine causal relationships and hypotheses involving multiple variables. SEM allows researchers to test and refine theoretical models by assessing how well the observed data align with the proposed relationships among variables. It’s a versatile approach that combines elements of factor analysis, path analysis, and regression analysis into a unified framework. Mediation analysis is a specific application of Structural Equation Modeling (SEM) used to understand the mechanisms through which an independent variable influences a dependent variable via one or more intermediate variables, known as mediators. In other words, mediation analysis helps researchers investigate the process or pathway by which an effect occurs.

### 4.1. Reliability, validity, and multicollinearity

Fornell and Larcker (1981) state that certain SEM analysis conditions must be met in order to evaluate the validity and reliability of a survey. Every latent variable in the study needs to have a Cronbach’s alpha value of 0.7 or above to be considered legitimate. According to Table 2, every latent variable’s Cronbach’s alpha value fits the criteria that support the validity of this study. Additionally, Average Variance Extracted (AVE) is the well-known reliability index. For dependability acceptance, Segars (1997) advised that the value of this index should be better than 0.5. The recommended ideals and norms are met by this index. As a result, the study model’s reliability is confirmed. Before evaluating the structural model, ensure no linear link exists there is no linear link between the elements. Variation inflation factor (VIF) values less than 5 were considered acceptable by Hair et al. (2019).

**Table 2:** Reliability, validity, and multicollinearity analysis.

Variables	Cronbach alpha	AVE	VIF
Entrepreneurial education	0.876	0.628	(3.07, 3.95)
Entrepreneurial mindset	0.711	0.729	(3.22, 4.88)
Entrepreneurial inspiration	0.709	0.652	(3.13, 3.92)
Entrepreneurial self-efficacy	0.817	0.558	(2.65, 3.44)
Entrepreneurial creativity	0.792	0.631	(3.87, 4.14)
Entrepreneurial intention	0.821	0.666	(3.87, 4.76)

According to Hair et al. (2011), the ideal discriminant validity is shown by the square root of the AVE of each construct (latent variable) being greater than its correlation coefficient values with other constructs. According to the study’s findings, which are presented in Table 3, the six variables of entrepreneurial education, entrepreneurial creativity, entrepreneurial mindset, entrepreneurial inspiration, entrepreneurial self-efficacy and entrepreneurial intention all had strong discriminant validity.

**Table 3:** Discriminant validity.

Variables	(1)	(2)	(3)	(4)	(5)	(6)
1-Entrepreneurial education	0.628					
2-Entrepreneurial mindset	0.534	0.729				
3-Entrepreneurial inspiration	0.601	0.472	0.652			
4-Entrepreneurial self-efficacy	0.432	0.532	0.321	0.558		
5-Entrepreneurial creativity	0.472	0.456	0.278	0.311	0.631	
6-Entrepreneurial intention	0.332	0.284	0.309	0.234	0.407	0.666

### 4.2. Structural model

We discovered that entrepreneurial education had a direct and significant positive effect on entrepreneurial intention (Beta = 0.411, C.R = 6.59). Consequently, H1 was approved. In addition, the results indicate that entrepreneurial education has a direct and significant positive effect on entrepreneurial inspiration (Beta = 0.476, C.R = 7.35). Therefore, H2 was confirmed. Entrepreneurial education had a direct positive and statistically significant effect on entrepreneurial mindset (Beta = 0.498, C.R = 7.99). As a result, H3 was approved. In addition, the results indicate that entrepreneurial mindset had a direct and significant positive influence on entrepreneurial intent (Beta = 0.501, C.R = 8.43). Additionally, H4 was supported. Moreover, we discovered that entrepreneurial creativity had a direct, positive, and statistically significant relationship with entrepreneurial intention (Beta = 0.408, C.R = 6.43). Therefore, H5 was approved. Moreover, we found that entrepreneurial inspiration had a direct correlation with entrepreneurial intention that was positive and statistically significant (Beta = 0.629, C.R = 9.53). As a result, proposition H6 was successful. We put the proposed hypotheses to the direct test, and the results are presented in Table 4.

**Table 4:** Direct estimations.

Hypotheses	Relationships	Standardized	Critical ratio (C.R)	*p*-value
		Estimates		
H1	EE → EINT	0.411**	6.59	<0.001
H2	EE → EINS	0.476**	7.35	<0.001
H3	EE → EM	0.498**	7.99	<0.001
H4	EM → EINT	0.501**	8.43	<0.001
H5	EC → EINT	0.408**	6.43	<0.001
H6	EINS → EINT	0.629**	9.53	<0.001

EE, entrepreneurial education; EM, entrepreneurial mindset; EINS, entrepreneurial inspiration; ES, entrepreneurial self-efficacy; EC, entrepreneurial creativity; EINT, entrepreneurial intention. Significant *p < 0.05, **p < 0.001.

In order to examine the indirect effect between the research variables, a bootstrap test was conducted with 5,000 bootstrap samples at 95% confidence intervals (CI) of the lower (LLCI) and upper bounds (ULCI). The method was calculated in accordance with the guidelines provided by Preacher and Hayes (2008). This analysis was conducted to determine the significance of the indirect effect. Table 5 illustrates that there is a positive indirect effect of both entrepreneurial mindset and entrepreneurial inspiration on the link between entrepreneurial education and entrepreneurial intention. Consequently, hypotheses H7 and H8 have been deemed acceptable. Furthermore, our study revealed that entrepreneurial self-efficacy demonstrated a significant and positive indirect impact on the association between entrepreneurial education, entrepreneurial inspiration, and entrepreneurial mindset with entrepreneurial intention. Therefore, the hypotheses H9, H10, and H11 were likewise deemed acceptable.

**Table 5:** Indirect estimations.

Hypotheses	Relationships	Standardized	(LLCI, ULCI)	*p*-value
		Estimates		
H7	EE → EM→ EINT	0.249	(0.198, 0.311)	0.001
H8	EE → EINS → EINT	0.299	(0.202, 0.327)	0.001
H9	EE → ES → EINT	0.170	(0.132, 0.209)	0.04
H10	EINS → ES → EINT	0.199	(0.125, 0.242)	0.01
H11	EM → ES → EINT	0.187	(0.138, 0.224)	0.02

EE, entrepreneurial education; EM, entrepreneurial mindset; EINS, entrepreneurial inspiration; ES, entrepreneurial self-efficacy; EC, entrepreneurial creativity; EINT, entrepreneurial intention. Significant *p < 0.05, **p < 0.001.

## 5. Discussion

The Chinese civilization dates back more than 5,000 years, making it one of the world’s oldest. In the course of its lengthy historical development, it has produced an outstanding traditional culture that survives to the present day and continues to influence the ideas and actions of all Chinese people. As a result of the influence of Confucianism, people in China place a high value on personal connections. This means that college students’ decisions on whether or not to pursue a career in entrepreneurship may be influenced by the recommendations of their parents and friends. In addition, China’s long-term and intimate social networks offer a useful learning platform where entrepreneurs can receive important business resources.

In regard to the first hypothesis, the findings suggest that entrepreneurial education had a positive and statistically significant influence on the intention to engage in entrepreneurial activity. This finding is comparable to those of earlier research scholars (Lv et al., 2021; Sun et al., 2023), who suggested that entrepreneurial education effectively stimulates students to become entrepreneurs. Lim et al. (2021) found that nursing students were more likely to become entrepreneurs if they had received entrepreneurial education. This finding’s justification is that entrepreneurship education gives students enough knowledge related to entrepreneurship that will serve as their manual for starting their own businesses. Through theory and practise, entrepreneurial education aims to improve students’ knowledge, preparedness, and entrepreneurial aptitude. Through hands-on experience, entrepreneurial education enables students to gain a wealth of knowledge and use it immediately. In addition, as part of the culture of entrepreneurship that exists in China, educational institutions make it possible for students to engage in conversation with established businesspeople in the hopes of gaining original concepts for forthcoming commercial endeavours. According to Yan et al. (2023), the importance of students receiving incentive to be entrepreneurial from both their teachers and their peers cannot be overstated.

According to the findings of this study, there is a significant connection between entrepreneurship education development of inspiration. The findings provide credence to earlier research conducted by Cui et al. (2021) and Wang et al. (2022), Entrepreneurial education and the development of a dynamic framework for research that is incorporated with entrepreneurship education. This framework will contribute to the investigation of how entrepreneurship education determinants influence students’ emotions and cognition, including inspiration and the subsequent attitude of being entrepreneurial. The aforementioned research conducted by Winkler et al. (2015) found that both academic and non-academic aspects of the curriculum, such as learning engagement or field experiences, can promote cognitive matters such as inspiration, entrepreneurial motivation, mindset, self-efficacy, and entrepreneurial intentions.

This study found that, in addition to entrepreneurial intention, entrepreneurship education plays a critical influence in determining students’ mindsets toward entrepreneurship. This result confirms the strong association between an entrepreneurial mindset and entrepreneurship education found by Handayati et al. (2020) and Sun et al. (2023). This finding also lends support to the theoretical contribution of social cognitive theory (Bandura, 1985), which posited that the interaction between cognition characteristics such as mentality and environmental is positively associated with the entrepreneurial goals of the student. Bandura’s argument was based on the observation that students who were exposed to an entrepreneurial environment were more likely to go on to start their own businesses. According to Yuan et al. (2020), social cognitive theory encourages students to develop an entrepreneurial mentality and activates their cognitive elements, both of which ultimately result in students taking entrepreneurial action. This has demonstrated that encouraging students to develop an entrepreneurial attitude encourages them to have more knowledge, experience, competence, and motivation. Students’ entrepreneurial conduct is influenced by their entrepreneurial mindset, which is developed by entrepreneurial education and its school-based activities. Because being an entrepreneur allows them to select from a variety of alternatives, students often choose this path as their first career. When they are able to empower all facets of resources, which is achieved by beginning to understand entrepreneurship in practical methods, they are satisfied. This demonstrates that students nowadays have a mindset of entrepreneurship and have thought about both the advantages and disadvantages of being an entrepreneur. In a similar vein, Iqbal et al. (2022) stated in a prior study that having a mindset of entrepreneurship helps students have a better understanding of a variety of outcomes and circumstances that are crucial for entrepreneurial studies.

We found that creativity has a favourable and statistically significant impact on entrepreneurial intent in relation to Hypothesis 4. This finding is in line with a number of earlier studies (Shi et al., 2020; Jiatong et al., 2021b; Ferreira-Neto et al., 2023) that discovered creative people are more inclined to pursue entrepreneurial careers. In the past, the discipline of creativity research was mostly concerned with economic organisations, such as businesses and institutions for research and design, and the research participants were typically executives from businesses or people involved in research and design. College students were used as the research subjects in this study to widen the scope of creativity research and to apply the creativity theory to the sphere of higher education. Second, there is a need for more research on the variables that affect an individual’s intention to start a business. Despite the fact that earlier studies focused heavily on the impact of human traits on entrepreneurial intention, creativity is still an easily overlooked personal component. In order to increase the influencing elements of entrepreneurial intent, this study presents creativity as a personal aspect. This supports the findings and theories of Pham and Le (2023) and Pham et al. (2023), according to which creativity can be seen as a valuable “raw material” that individuals own. By increasing entrepreneurship awareness and skills, such as opportunity identification, among other things, creativity can help people increase their entrepreneurial intention.

Regarding Hypothesis 3, the findings show that an entrepreneurial self-efficiency had a positive and significant influence on the students’ intention to engage in entrepreneurial activity. People with higher levels of self-efficacy have greater intents, and they also believe that they are more likely to accomplish positive outcomes by tracking a decided plan (Wang et al., 2023). This was discovered in studies that examined the direct impact of self-efficacy on entrepreneurial ambitions. In addition, business owners tend to be tenacious individuals who are self-assured on their chances of achievement and base their assurance in their abilities on their perception of their own self-efficacy. According to this viewpoint, there have been studies which confirm that higher levels of self-efficacy are positively connected to higher levels of entrepreneurial ambitions (Chien-Chi et al., 2020; Christensen et al., 2023). These studies were conducted by researchers in China and the United States. In addition, the interactions between inspiration, mindset, education, and self-efficacy have an effect on intention. Inspiration is one of the most important characteristics for entrepreneurs to have in order to be successful in their endeavors. Entrepreneurs are known for devising novel and resourceful methods to carry out a certain action or find a solution to a particular problem. In this regard, inspiration and mentality are what makes entrepreneurs recognize and develop alternative and potentially innovative approaches, assisting them in devising ways to act according to the drives of passion and self-efficacy, so altering their intentions to engage in entrepreneurial activity.

## 6. Conclusion

In this study, the researchers studied how several components of entrepreneurship, such as education, mindset, and creativity, affect the intention to start a company. Specifically, the researchers looked at how education, mentality, and creativity affect entrepreneurship. This study explores the ambitions of Chinese students to start their own enterprises and provides new insights into the background of the student sample from China. The sample was comprised of students studying in China. Within the scope of this investigation, a structural equation model was proposed, and with the assistance of SPSS and AMOS software, it was tested on the basis of the responses of 448 Chinese business students who were thought to have credibility.

The findings of this research indicate that having an entrepreneurial inspiration is associated with a greater likelihood of intending to launch one’s own company than either entrepreneurial self-efficiency or creativity. These findings suggest that an individual’s perception of their own self-efficacy as an entrepreneur favourably influences the relationship between entrepreneurial education, an entrepreneurial mindset, and creative capacity that is focused on entrepreneurial purpose.

On the basis of the findings of the study, we provided some useful recommendations for educational officials and educators. To begin, educators need to increase their ability and competency, particularly in reference to entrepreneurship courses. To encourage students’ creative potential, strategies like in-house training and enrolment in entrepreneurship courses are only two examples. In addition to emphasizing classroom teaching techniques, this would encourage the development of additional entrepreneurship curriculum activities. These are particularly effective at instilling an entrepreneurial spirit in students’ minds in the Chinese culture. This would be a constructive change.

In conclusion, the government ought to take steps to improve the entrepreneurial climate for college students. These steps include the establishment of a program to support social entrepreneurship, the provision of company funding, and the provision of free business spaces where students may easily launch their new ventures.

## 7. Implications and limitations

In light of the research results, we have provided a set of pragmatic recommendations for educators and policymakers. Educators enhance their proficiency and expertise, specifically in the realm of entrepreneurship education, through various means such as engaging in in-house training, participating in webinars focused on entrepreneurship, and implementing an entrepreneurship certification programme. Second, the institution has the potential to further boost the caliber of entrepreneurial education by broadening the range of instructional resources employed in entrepreneurship courses, thereby fostering the development of students’ creative abilities. This initiative would facilitate diverse learning opportunities, encompassing not only traditional classroom instruction but also supplementary entrepreneurship-focused curricular activities. These activities have demonstrated notable efficacy in cultivating entrepreneurial aspirations among Chinese students. Furthermore, in order to promote the development of entrepreneurship, it is imperative for university administrators to revise the curriculum of entrepreneurship courses by incorporating practical knowledge and skills from the field, rather than solely relying on traditional classroom instruction. Additionally, the university offers fundamental resources to students for the establishment of entrepreneurial ventures, such as company incubation centers and various forms of financial assistance.

While the findings to date contribute to the expansion of entrepreneurial intention theory, it is important to note that this study does have some limitations. First, a cross-sectional design was used for the nature of this study, and a self-administered questionnaire was used to collect the necessary data for analysis. This would be done with the assistance of a longitudinal research design in order to make a greater contribution to the field of entrepreneurship. Second, the students in this study were exclusively from Shandong Province, which limits the study’s capacity to be generalized to students from other Chinese regions and cities. Diverse geographical conditions in China may result in diverse entrepreneurial settings and resources, which may have the potential to influence entrepreneurial intention. This is because China is such a large country with a wealth of natural resources. As a result, subsequent studies ought to increase the sample size as well as the geographical area in order to make the research more applicable. The study specifically targeted university students enrolled in the business department as the population of interest. Subsequent investigations might potentially explore additional provinces within China or encompass a broader range of students, such as those attending vocational schools, as well as those pursuing studies in the fields of information technology and engineering.

## Data availability statement

The original contributions presented in the study are included in the article/supplementary materials, further inquiries can be directed to the corresponding author.

## Ethics statement

Ethical review and approval was not required for the study on human participants in accordance with the local legislation and institutional requirements. Written informed consent from the participants was not required to participate in this study in accordance with the national legislation and the institutional requirements.

## Author contributions

HJ and YL contributed to the conception and design of the study. YL, HJ, and KC organized the database, performed the statistical analysis, wrote the first draft of the manuscript, and wrote sections of the manuscript. All authors contributed to the article and approved the submitted version.

## Conflict of interest

The authors declare that the research was conducted in the absence of any commercial or financial relationships that could be construed as a potential conflict of interest.

## Publisher’s note

All claims expressed in this article are solely those of the authors and do not necessarily represent those of their affiliated organizations, or those of the publisher, the editors and the reviewers. Any product that may be evaluated in this article, or claim that may be made by its manufacturer, is not guaranteed or endorsed by the publisher.

## Figures and Tables

**Table:** 

Latent variable	Measures
Entrepreneurial education	I think that vocational students become entrepreneurs because of the entrepreneurial instruction they receive in school. The educational activities include entrepreneurship-related topics and give students the chance to launch their own businesses. I believe that educational activities could increase the opportunity for business. The classroom learning model offers the necessary information for entrepreneurship. The formal setting’s entrepreneurial education paradigm encourages original thought. Education in school develops entrepreneurship-related knowledge and abilities.
Entrepreneurial mindset	I’ve made the decision to pursue business prospect ideas through entrepreneurial activities. I have seen time allotment for business-related issues. I’ve examined if engaging in entrepreneurial activity is advantageous for me. I’ve thought about my financial options for engaging in entrepreneurial activities. I’ve considered both positive and negative emotions to entrepreneurial activities. I looked at potential opportunities and obstacles related to entrepreneurial activities.
Entrepreneurial intention	In the future, I’m going to start my own business. My professional ambition is to launch my own business. I’ll work hard to launch and manage my own business. I firmly believe that I will open a business in the future. I’ll do whatever to succeed as an entrepreneur. I firmly intend to launch a business someday.
Entrepreneurial creativity	I am good at coming up with original concepts. I frequently have fresh, useful ideas. I frequently support and champion others’ ideas. I excel at offering solutions that are novel. I’m good at coming up with original answers to issues. I frequently have fresh, original thoughts.
Entrepreneurial self-efficacy	I am confident in my ability to successfully commercialise concepts. I prefer to find unique solutions to challenges. I’m good at coming up with new ideas. I am open to trying new things. I am able to offer fresh comments and ideas. I’m accustomed to deliberating about my choices carefully. I excel at examining the outside world to identify opportunities and potential issues.
Entrepreneurial inspiration	Over the past year, I’ve put a lot of effort into my creativity and entrepreneurship. My consideration of starting a business is aided by my schooling in entrepreneurship. I concur that having my own business gives me a fantastic sense of accomplishment. There are some entrepreneurial inputs that significantly altered my “heart and mind” and led me to explore starting a business. I look forward to exploring new possibilities in this atmosphere.
